# Alpha-2-macroglobulin as a novel diagnostic biomarker for human bladder cancer in urinary extracellular vesicles

**DOI:** 10.3389/fonc.2022.976407

**Published:** 2022-09-13

**Authors:** Jisu Lee, Hyun Sik Park, Seung Ro Han, Yun Hee Kang, Ji Young Mun, Dong Wook Shin, Hyun-Woo Oh, Yoon-Kyoung Cho, Myung-Shin Lee, Jinsung Park

**Affiliations:** ^1^ Department of Microbiology and Immunology, Eulji University School of Medicine, Daejeon, South Korea; ^2^ Department of Urology, Eulji University Hospital, Eulji University School of Medicine, Daejeon, South Korea; ^3^ Eulji Biomedical Science Research Institute, Eulji University School of Medicine, Daejeon, South Korea; ^4^ Neural Circuit Research Group, Korea Brain Research Institute, Daegu, South Korea; ^5^ Department of Family Medicine/Supportive Care Center, Samsung Medical Center, Sungkyunkwan University School of Medicine, Seoul, South Korea; ^6^ Core Facility Management Center, Korea Research Institute of Bioscience and Biotechnology, Daejeon, South Korea; ^7^ Department of Biomedical Engineering, Ulsan National Institute of Science and Technology (UNIST), Ulsan, South Korea; ^8^ Center for Soft and Living Matter, Institute for Basic Science (IBS), Ulsan, South Korea; ^9^ Department of Urology, Uijeongbu Eulji Medical Center, Eulji University, Uijeongbu-si, South Korea

**Keywords:** alpha-2-macroglobulin, bladder cancer, extracellular vesicles (EVs), urine, biomarker

## Abstract

Extracellular vesicles (EVs) derived from urine are promising tools for the diagnosis of urogenital cancers. Urinary EVs (uEVs) are considered potential biomarkers for bladder cancer (BC) because urine is in direct contact with the BC tumor microenvironment and thus reflects the current state of the disease. However, challenges associated with the effective isolation and analysis of uEVs complicate the clinical detection of uEV-associated protein biomarkers. Herein, we identified uEV-derived alpha-2-macroglobulin (a2M) as a novel diagnostic biomarker for BC through comparative analysis of uEVs obtained from patients with BC pre- and post-operation using an antibody array. Furthermore, enzyme-linked immunosorbent assay of uEVs isolated from patients with BC (n=60) and non-cancer control subjects (n=23) validated the significant upregulation of a2M expression in patient uEVs (p<0.0001). There was no significant difference in whole urine a2M levels between patients with BC and controls (p=0.317). We observed that compared to classical differential centrifugation, ExoDisc, a centrifugal microfluidic tangential flow filtration device, was a significantly more effective separation method for uEV protein analysis. We expect that our approach for EV analysis will provide an efficient route for the identification of clinically meaningful uEV-based biomarkers for cancer diagnosis.

## Introduction

Bladder cancer (BC) is the second most common genitourinary tract malignancy worldwide (1). Currently, BC is diagnosed *via* cystoscopy and urine cytology. However, cystoscopy is invasive as well as painful (2), and it may overlook carcinoma-*in-situ* or flat lesions (3). While urine cytology is non-invasive, it has low sensitivity, especially for low-stage/low-grade BC (4). To overcome the limitations of current diagnostic approaches, novel urine-derived BC biomarkers have been identified (4, 5). However, none of these have exhibited superior diagnostic accuracy when compared to cystoscopy and urine cytology.

Extracellular vesicles (EVs) are membrane-bound organelles secreted by cells and contain different types of molecular cargo (e.g., RNA, proteins, and metabolites) (6). EVs are released into the extracellular space and can be found in various types of bodily fluids, including the blood, urine, cerebrospinal fluid, and saliva. As mediators of intercellular communication, EVs regulate a wide range physiological responses and pathological processes (7, 8). Cancer cells secrete more EVs than normal, and tumor cell-derived EVs are involved in cancer progression (9, 10). Since EVs isolated from urological cancers contain cancer-specific proteins and nucleic acids, they may hold potential as cancer biomarkers, allowing for the non-invasive diagnosis and monitoring of urological malignancies (11–15). Following the discovery of urinary EVs (uEVs) in human urine in 2004 (16), several studies have been performed for the identification of uEV biomarkers in various renal, urogenital, and systemic diseases (17, 18).

In theory, since urine is in direct contact with the urothelium, uEVs may reflect the status of the latter, carrying the molecular cargo derived from urothelial cells and thus indicating the presence or absence of BC. Several previous studies have suggested the potential of uEV-based markers for BC diagnosis. For example, a previous study reported that EGF-like repeat and discoidin I-like domain 3 levels from uEVs of patients with high-grade BC were significantly higher than those from the uEVs of healthy controls (19), and uEV-derived periostin was associated with the prognosis of muscle-invasive BC (20). Other studies have suggested the potential of uEV-derived lncRNA (HOX transcript antisense RNA) (21) and miRNA as biomarkers in BC (22).

Although uEVs hold promise for BC diagnosis, only a few studies have clinically validated uEV markers. In order to identify clinically accurate uEV-derived protein biomarkers for BC, we integrated a number of approaches for comprehensive EV analysis. First, we employed uEV isolation *via* ExoDisc, a centrifugal microfluidic tangential flow filtration system, in order to obtain sufficient uEV-derived protein (23, 24). We subjected uEVs from BC patients to antibody array-based proteome analysis, as opposed to analyzing the culture medium from BC cell lines, as mostly done in previous related studies (19–22). Further, we compared pre- and post-operative uEV proteomes of the same patients in order to avoid the inter-individual variability among urine specimens reported in previous studies (25–27). Finally, we validated the putative diagnostic biomarker in a real BC patient cohort. The combination of these approaches represents a practical approach for the discovery of novel uEV biomarkers for non-invasive BC diagnosis.

Alpha-2-macroglobulin (a2M) is a large protein found in the blood. Its elevated levels are seen in clinical conditions such as chronic liver disease, inflammatory joint diseases, multiple sclerosis, and nephrotic syndrome. Decreased levels are seen in rheumatoid arthritis in women (28). However, changes of the a2M in EVs have not been studied much in diseases yet. In this study, to the best of our knowledge, a2M in uEVs was identified as a biomarker in bladder cancer for the first time.

## Materials and methods

### Human samples and data collection

Urine samples were obtained from study participants at Eulji University Hospital (Daejeon, Republic of Korea) between July 2018 and October 2020. In collecting BC urine samples, no predefined selection criteria were present. If the patients agreed to urine sample collection and provided informed consent to the study protocol, urine samples of them were obtained and stored in the biobank of our hospital. To identify optimal EV isolation methods, urine samples from one non-cancer control and one BC patient were collected. Further, in order to screen candidate uEV protein biomarkers, pre- and post-operative urine samples from four BC patients (two high-grade, two low-grade; total eight samples) were analyzed using an antibody array. Moreover, to assess the potential of several candidate uEV protein biomarkers as a BC diagnostic marker, urine samples of 20 BC patients and 10 non-cancer controls collected at an early stage of the study (July 2018 to March 2019) were used for a pilot test using an enzyme-linked immunosorbent assay (ELISA). Finally, 83 urine samples (23 non-cancer control, 28 low-grade BC, 32 high-grade BC) were collected to validate the diagnostic value of the selected uEV protein biomarker through ELISA for the final study. First-morning urine samples of control or BC patients were obtained before transurethral resection as well as at the post-operative 3-month follow-up cystoscopy. Supernatants were separated from urine samples through centrifugation at 2,000 *g* for 10 min, filtered through a 0.8 μm syringe filter, and immediately stored at -80°C until use (29). For non-cancer controls, urine samples were collected at the outpatient clinic and processed in the same manner. BC was staged based on the 2010 TNM staging system (30) and graded according to the 2004 World Health Organization grading system (31). Procedures involving urine sample collection and analysis for this study were approved by the Institutional Review Board of Eulji University Hospital (No. 2018-07-010 and 2019-05-027-002) and conducted according to the principles outlined in the Declaration of Helsinki. All subjects were informed regarding the purpose of the experiment and provided written informed consent before participating in the study.

### Separation and analysis of EVs

After obtaining the two urine samples (one from a non-cancer control and one from a BC patient), each sample was divided into three equal volumes and subjected to three different separation methods, namely differential centrifugation, ExoLutE^®^ Urine kit (Rosseta Exosome, Seongnam, South Korea), and ExoDisc for urine (LabSpinner ExoDiscovery, Seoul, South Korea) (23). Differential centrifugation was performed as previously described, with minor modifications (32, 33). Briefly, the collected urine was centrifuged at 2,000 × *g* for 10 min and filtered through a 0.45 μm filter in order to remove debris. The prepared urine was centrifuged at 10,000 × *g* for 30 min and then at 100,000 × *g* for 60 min. The pellet was dissolved in 0.22 μm-filtered cold PBS for EV collection. For isolation using the ExoLutE kit, debris was removed from the urine *via* centrifugation and filtration. The prepared urine was pre-cleared with Sol U supplied with the kit (pre-clearing). Crude EVs were then precipitated in Sol A, B, and C provided with the kit (enrichment). The dissolved pellet was processed in a spin-based size-exclusion column in order to separate the EVs (purification). For the ExoDisc method, the urine was centrifuged at 2,000 × *g* for 10 min, and the supernatant was filtered through a 0.22 μm syringe filter. For priming, PBS was added to the filter chamber and centrifuged in a LabSpinner centrifuge for 5 min in order to activate the filter. The prepared urine was then transferred to filter chambers and centrifuged for 5–30 min in order to separate the EVs for enrichment. Finally, the collected EVs were washed twice by adding PBS to the filter chambers, and the solution was centrifuged in ExoDiscovery. The number and size distribution of microparticles in the EV preparations were analyzed using the nanoparticle tracking analyzer ZetaView (Particle Metrix GmbH, Meerbusch, Germany) as previously described (34). EV preparations were diluted in PBS and passed through 0.8 μm filters before analysis. The analysis parameters were as follows: maximum area of 1,000, minimum area of 10, minimum brightness of 25, sensitivity of 75, shutter of 100, and temperature of 25°C. uEVs were assessed using TEM as described previously (35). In order to observe EVs, purified vesicles were applied to a freshly charged carbon-formvar-coated grid and stained with 1% uranyl acetate solution for 1 min. The stained vesicles were observed at 200 kV under a Tecnai G2 Transmission Electron Microscope (Thermo, Waltham, MA).

### Protein-based EV quantification and analysis of EV proteins

Protein-based quantification of isolated EVs was performed using a micro bicinchoninic acid (BCA) assay kit (Thermo) immediately after isolation. Subsequently, equal volumes of EVs isolated *via* different methods were denatured using 5× sample buffer without dithiothreitol (DTT) at 95°C for 10 min and then resolved on a 10–12% SDS-acrylamide gel *via* electrophoresis. Resolved proteins were transferred onto nitrocellulose membranes (GE Healthcare, Uppsala, Sweden), which were then blocked *via* incubation in 5% skimmed milk with 0.1% Tween-20 buffer in order to minimize the non-specific binding of antibodies. Blocked membranes were then treated with primary antibodies overnight at 4°C, washed three times with 1× Tris-buffered saline with 0.1% Tween-20 (TBST) buffer, and incubated with an HRP-conjugated secondary antibody for 1 h at room temperature. Unbound antibodies were removed by washing with 1× TBST buffer, and immunolabeled proteins were visualized using the West Femto Maximum Sensitivity Substrate Kit (Thermo) and an Amersham ImageQuant 800 system (GE Healthcare). Mouse monoclonal anti-beta-tubulin (AC021, ABclonal Technology, Wuhan, China), mouse monoclonal anti-CD81 (454720, R&D Systems, Minneapolis, MN), mouse monoclonal anti-CD63 (sc-5275, Santa Cruz Biotechnology, Santa Cruz, CA), anti-CD9 (ab236630, Abcam, Waltham, MA), anti-ALIX (sc-53540, Santa Cruz Biotechnology, CA), anti-TSG101 (bs-1365R, BIOSS, Woburn, MA), anti-HSP70 (bs-0244R, BIOSS), anti-a2M (sc-390544, Santa Cruz Biotechnology), HRP-conjugated goat anti-mouse IgG antibody (A90-116P, Bethyl Laboratories, Montgomery, TX), and HRP-conjugated goat anti-rabbit IgG antibody (A120-101P, Bethyl Laboratories) were used. The same quantity of uEV protein (5 µg per array) was used for Proteome Profiler antibody arrays, including a human soluble receptor antibody array and a non-hematopoietic panel (R&D Systems). The assay was performed according to the manufacturer’s instructions. Each spot signal was visualized using an Amersham ImageQuant 800 system (GE Healthcare), and signals were quantified using Quick Spots image analysis software (R&D Systems). The levels of cadherin-13, clusterin, and a2M in uEVs were analyzed using a human cadherin-13 ELISA kit (RayBiotech, Peachtree Corners, GA), human clusterin DuoSet ELISA kit (R&D Systems), and human alpha 2-macroglobulin DuoSet ELISA kit (R&D Systems) according to the manufacturers’ instructions. After separation and quantification of uEVs, 0.5 µg of EVs was applied to each well.

### Cell culture and preparation of cell lysate

BC cell lines (RT4, 5637, TCC-SUP, HT-1197, HT-1376, and T24) were obtained from the Korean Cell Line Bank (Seoul, South Korea) or American Type Culture Collection (ATCC, Rockville, MD). All cells were culture in Dulbecco’s modified Eagle’s medium (DMEM) supplemented with 10% fetal bovine serum (Serum source international, Charlotte, NC). The cells were maintained in a humidified atmosphere of 5% CO_2_ at 37°C. Whole cell proteins were isolated using 1 × SDS buffer containing 62.5 mM Tris-HCL at pH 6.8, 2% w/v SDS, 10% v/v glycerol, 50 mM dithiothreitol, and 0.01% w/v bromophenol blue.

### Immunohistochemistry for a2M in BC tissues

Human BC specimens were obtained from the Eulji University Hospital. Paraffin-embedded tumor sections were incubated with the anti-a2M antibody (1:200, sc-390544, Santa Cruz Biotechnology) overnight at 4°C after blocking with normal horse serum (Vector Laboratories, Burlingame, CA) for 1 h at room temperature. The sections were then incubated for 15 min with an Amplifier antibody (goat anti-mouse IgG, MP-7602, Vector Laboratories), followed by incubation with ImmPRESS Excel Reagent (horse anti-goat IgG, MP-7602, Vector Laboratories) for 30 min. Immunoreactivity was visualized with 3,3-diaminobenzidine (DAB), and Mayer’s hematoxylin was used for counterstaining. Finally, the slides were observed under an optical microscope (CX23, Olympus, Tokyo, Japan), and images were captured with an eXcope T500 camera (DIXI Science, South Korea).

### Iodixanol density gradient fractionation

Iodixanol density gradient fractionation was performed as previously described, with modifications (27). Iodixanol (OptiPrep) density gradient medium (BioVision Inc., Milpitas, CA) was prepared in ice-cold PBS immediately before use in order to generate discontinuous step (12, 18, 24, 30, and 36) gradients. EVs were resuspended in PBS and mixed with ice-cold iodixanol/PBS in order to obtain the final 36% iodixanol solution. The suspension was added to the bottom of a centrifugation tube, and solutions of descending concentrations of iodixanol in PBS were carefully layered on top, yielding a complete gradient. The bottom-loaded 12–36% gradients were subjected to ultracentrifugation at 120,000 × g for 15 h at 4°C. Twelve individual fractions of 1 ml were collected from the top of the gradient. For immunoblotting, each individual fraction with equal volumes of EVs was denatured using 5× SDS-PAGE sample buffer (250 mM Tris-HCl, pH 6.8, 0.5 M DTT, 10% SDS, 50% Glycerol, 0.2% Bromophenol blue) with or without 0.5 M DTT at 95°C for 10 min and then resolved on a 6–12% SDS-acrylamide gel *via* electrophoresis.

### Immunoprecipitation analysis

Immunoprecipitation was performed as previously described, with minor modifications (36, 37). Briefly, EVs were incubated with a rabbit polyclonal anti-a2M antibody (A1573, ABclonal Technology) and IgG from rabbit serum (I5006, Sigma-Aldrich, St. Louis, MO) overnight at 4°C. The antigen-antibody complexes were precipitated with Pierce protein A/G Agarose (#20421, Thermo) for 2 h at room temperature. The immunoprecipitated complexes were cleared using 1× PBS and analyzed *via* western blotting, as described as described in earlier.

### Statistical analysis

Each experiment was independently performed at least three times, and representative results are shown. Results are presented as the mean ± standard deviation (SD). One-way ANOVA, two-tailed Student’s *t*-test, and paired sample *t*-test were used to assess the significance of differences between groups and pre-versus post-operative expression, respectively. A receiver operating characteristic (ROC) curve was used to evaluate the ability of a uEV marker to determine BC status. Sensitivity, specificity, and Youden index (sensitivity +specificity - 1) × 100% were calculated to determine the optimal cut-off. Statistical analyses were conducted using Stata version 14.0 (StataCorp., Houston, TX) and Prism (GraphPad Software, San Diego, CA). Statistical significance at *p* values of <0.05 and <0.01 is indicated by * and **, respectively.

## Results

### Evaluation of methods for EV isolation from human urine

**Figure 1:**
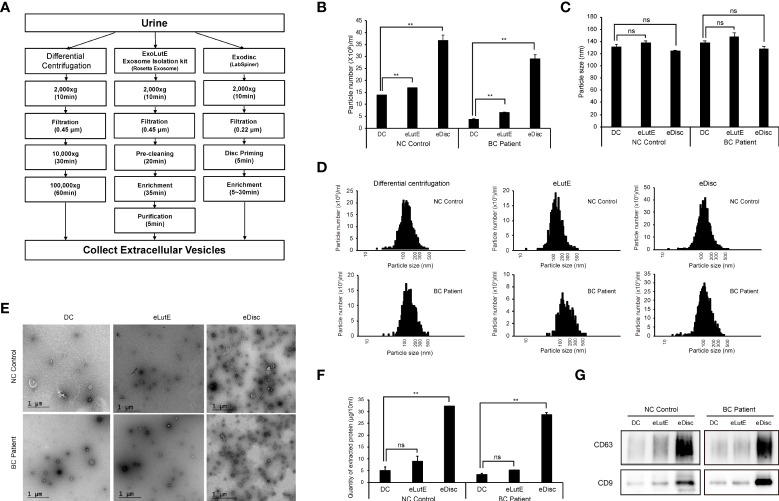
Comparison of the urinary EV separation efficiency between three different methods. Extracellular vesicles (EVs) were separated *via* three different methods, namely differential centrifugation, the ExoLutE exosome isolation kit, and ExoDisc. Separation efficiencies were then analyzed and compared. **(A)** Schematic summary of the EV separation methods. Human urine from one non-cancer control or bladder cancer patient was divided into three equal volumes (10 ml each), and each was subjected to a different isolation method. **(B–D)** Nanoparticle tracking analysis (NTA) of EVs separated through each method. The number **(B)**, size **(C)**, and size distribution **(D)** of EV particles separated *via* each method were determined through NTA. NC control: EVs isolated from non-cancer control, BC Patient: EVs isolated from bladder cancer patient. DC: EVs separated using differential centrifugation. eLutE: EVs separated using the ExoLutE exosome isolation kit. eDisc: EVs separated using ExoDisc from LabSpinner. Data are presented as the mean ± SD, n = 3, *p<0.05, **p<0.01, ns: not significant. **(E)** Transmission electron microscopy (TEM) images of separated EVs. **(F)** Quantification of protein in EV particles separated *via* each method. EV protein was quantified using the micro bicinchoninic acid (BCA) protein assay. Data are presented as the mean ± SD of three independent experiments, **p<0.01, ns, not significant. **(G)** Western blot analysis of CD63 and CD9 expression in EV particles separated *via* each method.

The isolation of sufficient EVs from urine is the most critical preliminary step for the analysis of uEV protein. In order to determine the optimal EV isolation method, EVs were isolated from urine samples of a control subject or BC patient using three different methods (**Figure 1A**). These included the standard differential ultracentrifugation method, ExoLutE Urine (a multistep combined exosome isolation kit involving size-exclusion chromatography), and ExoDisc (a microfluidic 20 nm size-selective nanofilter-based isolation method). The number and size distribution of EV particles isolated from the urine samples were analyzed *via* NTA. There was a significant difference in EV particle numbers between methods (**Figure 1B**). Differential centrifugation yielded the lowest number of particles, 1.3- to 9.4-fold lower than numbers obtained *via* the other two methods. In NTA, the median size distribution ranged from 120 nm to 140 nm in diameter, and the average particle size was not significantly different between methods (**Figure 1C**). Overall, a wide range of particle sizes from 20 ~ 500 nm was observed, with no significant difference in size distribution between methods (**Figure 1D**). EVs were present in all samples, as observed *via* TEM. However, more EVs were present in the samples obtained using ExoDisc, which was consistent with the particle numbers determined *via* NTA (**Figure 1E**). To compare the total protein quantity in uEVs isolated through different methods, we employed micro BCA assays (**Figure 1F**). EVs separated through differential centrifugation contained the lowest amount of protein. The EV fraction separated *via* ExoDisc contained significantly greater amounts of protein than EVs obtained *via* differential centrifugation (p<0.01). To verify the quantity of EV-derived protein, the same volume of protein lysate from separated EVs was subjected to western blot analysis (**Figure 1G**). Significantly larger quantities of CD63 and CD9, well-known EV markers, were detected in EVs obtained *via* the ExoDisc method than in EVs obtained *via* other methods. These results indicated that ExoDisc yielded greater amounts of uEV protein than the other tested methods. Therefore, the ExoDisc method was adopted for urinary EV separation in further experiments.

### Proteome analysis of uEVs from BC patients

**Figure 2:**
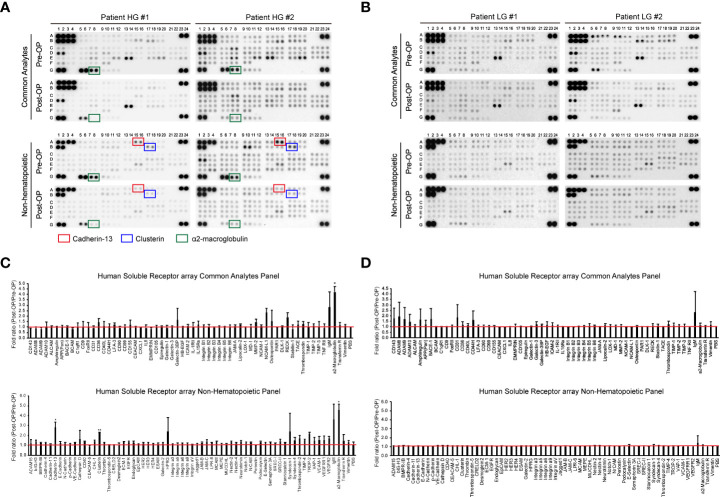
Screening of bladder cancer-associated EV proteins. Urine was collected from bladder cancer patients on the day before surgical intervention (Pre-OP) and at follow-up three months after surgery (Post-OP). EVs were separated from the collected urine using ExoDisc. After protein quantification with the micro BCA assay, 5 µg of EVs was subjected to a Proteome Profiler array for human soluble receptors. **(A, B)** Results from the protein array analysis of urinary EVs from high-grade bladder cancer patients **(A)** and low-grade bladder cancer patients **(B)**. Pre-OP and Post-OP: EVs from a pre- or post-operative bladder cancer patient urine. HG and LG: urinary EVs from high-grade (HG) or low-grade (LG) bladder cancer patients. Red, blue, and green boxes indicate cadherin-13, clusterin, and a2M, respectively. **(C, D)** Data analysis of antibody array results with urinary EVs from high-grade **(C)** or low-grade **(D)** bladder cancer patients. The fold ratio for the signal value of each protein (Pre-OP/Post-OP) is presented on the Y-axis. PBS was used as a negative control, and the calculated value for PBS is indicated by a red line. Data are presented as the mean ± SD, n = 2, *p<0.05.

Previous studies have identified uEV-derived disease-specific biomarkers using mass spectrometry-based proteomics, which allows the characterization of thousands of proteins present in small samples (16, 38–40). However, since the ultimate purpose of this study was to identify novel urinary EV protein biomarkers, which should be detectable *via* antibody-based methods, such as ELISA, it was determined that an antibody-based protein array would be more suitable than mass spectrometry-based proteomics. Thus, we used a Proteome Profiler antibody array for human soluble receptors, which allows for the analysis of 119 different receptors, as molecules present on the EV surface may be the best candidates for ELISA-based detection. Basic information of the four patients who were included in the protein array analyses for uEV biomarker screening before and after transurethral surgery is summarized in **Table 1**. Urine was collected from each BC patient before surgery (the day of transurethral resection of the bladder tumor) and 3 months after surgery (before follow-up cystoscopy, with no tumor recurrence confirmed). EVs were separated from 4 ml of urine using the ExoDisc method, and 5 μg of EV protein was used for proteome analysis (**Supplementary Tables 1** and 2). Antibody array data are presented in **Figure 2**. The analysis of uEVs from two high-grade BC patients revealed significant differences in the abundance of three proteins in uEVs, namely cadherin-13, clusterin, and a2M, before and after surgery (**Figures 2A** **, C**). The analysis of uEVs from patients with low-grade tumors did not reveal any significant differentially expressed proteins (**Figures 2B** **, D**).

**Table 1:** Clinical parameters of patients used for screening urinary EV-in the protein array after transurethral surgery.

	Patient HG#1	Patient HG#2	Patient LG#1	Patient LG#2
Pathology	T1, high grade & CIS	T1, high grade & CIS	Ta, Low grade	Ta, Low grade
Primary vs. Recurrent	Primary	Primary	Recurrent	Primary
Multiplicity	Single	Multiple	Multiple	Single
Pre-op. hematuria on urinalysis	Present	Present	Present	Present
Post-op. BCG instillation	Yes	Yes	Yes	No
Post-op. 3-mo hematuria on urinalysis	Absent	Absent	Absent	Absent

### Validation for a2M as a BC diagnostic biomarker in patient uEVs

**Figure 3:**
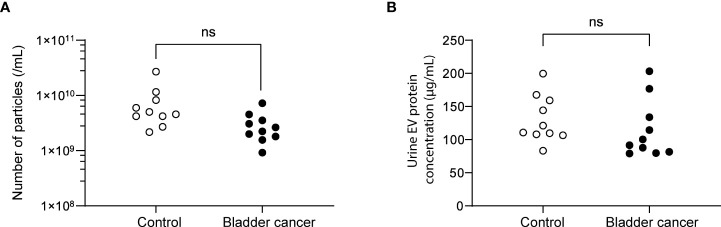
Analysis of the particle number and total proteins of uEVs from control and bladder cancer patients. **(A)** the particle numbers of uEVs were analyzed by a nanoparticle tracking analyzer, ZetaView. **(B)** total protein concentrations were analyzed by micro BCA. ns, not significant.

**Figure 4:**
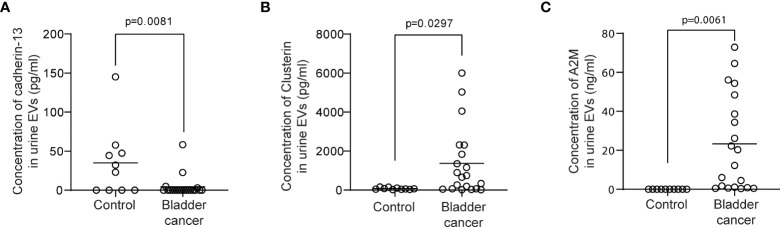
ELISA for clusterin, cadherin-13, and a2M in uEVs from non-cancer controls and BC patients. uEVs (0.5 μg/well) from 10 non-cancer controls and 20 BC patients were applied to ELISA for clusterin **(A)**, cadherin-13 **(B)**, and a2M **(C)**. Mean values of each group were indicated as lines.

**Figure 5:**
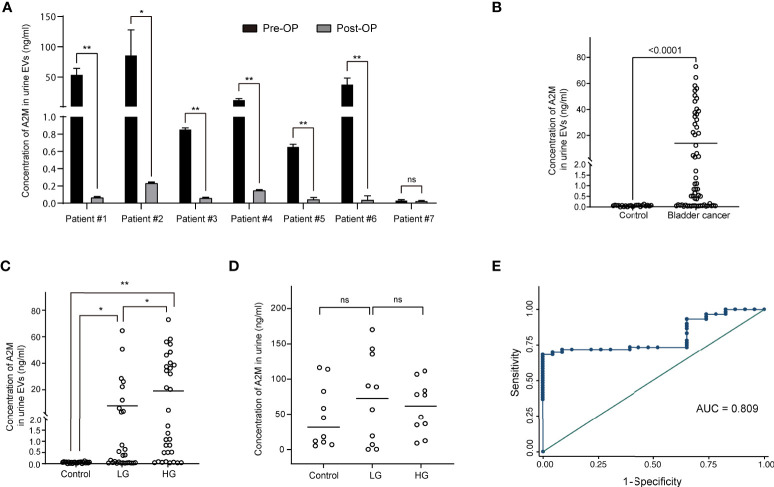
Clinical validation of a2M as a bladder cancer biomarker. After separating EVs from urine using ExoDisc, EVs (0.5 µg per sample) were subjected to ELISA for a2M. **(A)** Pre- and post-operative changes in urine EV a2M levels from seven individual cancer patients. a2M levels in urinary EVs were analyzed *via* ELISA before and after surgery in randomly selected bladder cancer patients. Paired sample t-test, *p<0.05, **p<0.01, ns: not significant. **(B)** Quantification of a2M in urinary EVs from non-cancer controls (N=23) and bladder cancer patients (N=60). Student’s t-test. **p<0.01. **(C)** Analysis for a2M in urinary EVs from non-cancer controls (N=23), patients with low-grade bladder cancer (N=28), and patients with high-grade bladder cancer (N=32). One-way ANOVA test. *p<0.05, **p<0.01. **(D)** Analysis for a2M in urine from non-cancer controls (N=10), low-grade (N=10), and high-grade bladder cancer patients (N=10). One-way ANOVA test. ns: not significant. **(E)** ROC curve analysis for the discrimination between BC patients and non-cancer controls based on uEV a2M expression levels as per ELISA. Area under ROC curve = 0.809.

Urine was collected from 83 patients (23 control subjects and 60 BC patients). The baseline characteristics of the clinical validation cohort for the expression of uEV target proteins are presented in **Table 2**. The control group included patients diagnosed with benign prostatic hyperplasia, urinary stones with hematuria, and cystitis with hematuria. For the three potential biomarker proteins identified in the protein array, we preliminarily analyzed their expression in uEVs from randomly selected 10 non-cancer controls and 20 BC patients. After separating EVs from each urine sample *via* the ExoDisc method, particle number and total protein of EVs in 10 samples per group were analyzed using ZetaView and micro BCA, respectively (**Figure 3**). There was no significant difference between the control and cancer groups. However, we observed that the difference in the number of particles was greater (up to 11.8 times) than the quantity of protein (up to 2.8 times) in each sample. Furthermores, much more amount of EVs was required for nanoparticle tracking analysis than micro BCA. Therefore, we decided to apply the quantity of proteins to the normalization and 0.5 µg of EVs per sample was subjected to ELISA for analysis of the three biomarkers. Among the three analyzed proteins, a2M exhibited the most significant difference between non-cancer control- and BC patient-derived uEVs (**Figure 4**), and thus a2M was selected for the final validation study. To determine the correlation between BC and a2M levels in uEVs, uEV a2M levels were determined for seven BC patients for whom pre-and postoperative urine EVs were obtained *via* ELISA (**Figure 5A**). For most of the analyzed patients, uEV-derived a2M level was significantly lower after surgery. We further validated its clinical significance by analyzing preoperative urine samples from all patients *via* ELISA. While there were few detectable signals for uEV a2M expression in the control group, a2M expression in uEVs from BC patients was significantly higher (**Figure 5B**, p<0.0001). The uEV a2M expression in the high-grade BC patient group was significantly higher than that in uEVs from low-grade BC patients (**Figure 5C**). In order to determine whether a2M level in the whole urine was significantly increased in BC patients, total urine a2M levels of BC patients and controls were analyzed *via* ELISA. Interestingly, a2M levels were detected in the whole urine samples of controls, with no significant difference compared to those in BC patients (**Figure 5D**). ROC curve analysis of the data in **Figure 5B** revealed good diagnostic performance, yielding an area under the curve of 0.809 (**Figure 5E**). The diagnostic performance of uEV a2M expression in discriminating BC patients from controls is summarized in **Table 3**. uEV a2M expression with a cut-off of 0.035 (highest Youden index) robustly discriminated BC patients from controls, with a sensitivity of 93.3%, a specificity of 34.8%, and a higher Youden index than that observed *via* urine cytology (**Table 3A**). Notably, among 60 BC patients, 47 were positive for uEV a2M (value >0.035), while their urine cytology results were negative, indicating that 78.3% of BC patients were identified only based on uEV a2M expression (**Table 3B**). Taken together, these results highlight the potential of uEV-derived a2M as a biomarker for BC diagnosis.

**Table 2:** Demographic characteristics of subjects for the analysis of a2M through ELISA.

	BC cases	Non-cancer controls
No. of patients	60	23
		(BPH: 17; urinary stone: 3; cystitis: 3)
Mean age (y) ± SD	66.77 ± 11.56	60.35 ± 7.40
Gender		
Male	55	21
Female	5	2
Tumor grade		
Low	28	
High	32	
T stage		
Ta	30	
CIS	5	
T1	22	
T2	3	
Hematuria on urinalysis		
Present	56	12
Absent	4	11
Cytology		
Negative	51	23
Positive	9	0

BC, bladder cancer; BPH, benign prostatic hyperplasia; a2M, alpha 2-macroglobulin; ELISA, enzyme-linked immunosorbent assay.

**Table 3:** Diagnostic performance of uEV a2M expression (based on ELISA) for discriminating between BC patients and non-cancer controls.

A. Sensitivity, specificity, AUC, and Youden index according to various cut-offs of a2M levels. Data of urine cytology are also shown as a reference.						
Cut-off	Sensitivity (%)		Specificity (%)		AUC	Youden index
0.025	96.7%		17.4%		0.57	14.1%
0.03	95.0%		26.1%		0.61	21.1%
0.035	93.3%		34.8%		0.64	28.1%
0.04	86.7%		34.8%		0.61	21.5%
0.045	76.7%		34.8%		0.56	11.5%
0.05	75.0%		34.8%		0.55	9.8%
Urine cytology	15.0%		100.0%		0.58	15.0%
B. Summary of a2M status with a cut-off of 0.035 (highest Youden index) and urine cytology results in the validation cohort.						
**Group**	**a2M expression status**	**urine cytology**	**clinical meaning**	**N**	**a2M ELISA, mean (SD)**	**a2M ELISA value, range**
Bladder cancer	negative	negative	both miss	4	0.026 (0.004)	0.022–0.031
(N=60)	positive	negative	Ucyt miss	47	10.9 (18.2)	0.035–64.55
	negative	positive	a2M miss	0	NA	NA
	positive	positive	both detect	9	35.3 (25.1)	0.058–72.91
Non-cancer control	negative	negative	both true	8	0.018 (0.014)	0–0.034
(N=23)	positive	negative	a2M false positive	15	0.07 (0.016)	0.06–0.12
	negative	positive	Ucyt false positive	0	NA	NA
	positive	positive	both false	0	NA	NA

Ucyt, urine cytology; uEV, urinary extracellular vesicle; a2M, alpha 2-macroglobulin; ELISA, enzyme-linked immunosorbent assay; BC, bladder cancer.

### a2M from BC is co-fractionated with common EV markers

**Figure 6:**
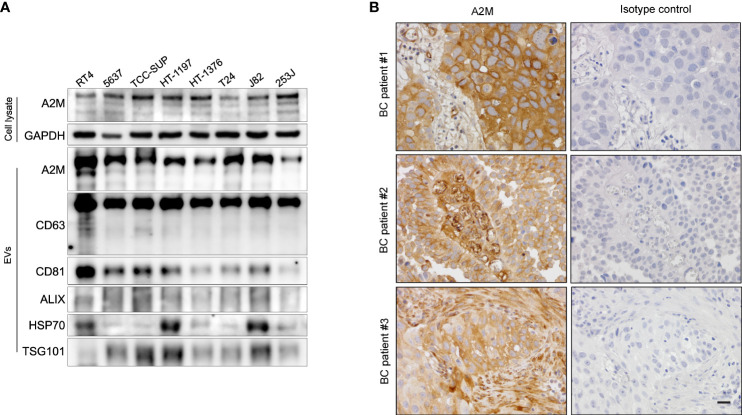
Expression of a2M in bladder cancer cell lines and tissues. **(A)** Western blot analysis of a2M in bladder cancer cell lines. **(B)** Immunohistochemistry for a2M in BC tissues. Representative a2M immunohistochemical images in BC tissues. a2M immunostaining showed high cytoplasmic expression in tumor cells with atypical larger nuclei of invasive urothelial carcinoma in BC patient #1. In BC patient #2, a2M expression exhibited a significant increase in the central fibrovascular core of the papillary tumor. a2M was expressed in the fibrous tumor stroma of BC patient #3. Right panel: Negative images using isotype control in bladder cancer tissues. Magnification, ×400. Scale bars, 20 μm.

**Figure 7:**
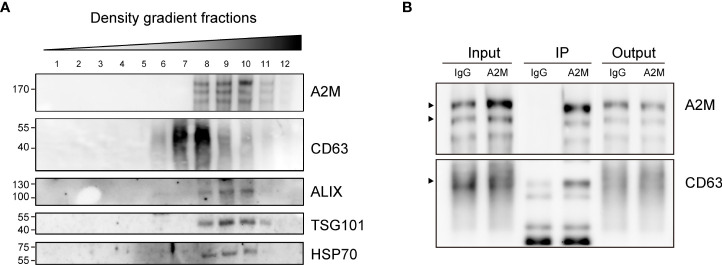
Co-fractionation of a2M with EV markers. **(A)** Density gradient fractionation of urinary EVs from bladder cancer patient. After flotation of the sample in high-resolution iodixanol gradients, equal volumes of each fraction were loaded on SDS-PAGE gels, and membranes were blotted with the indicated antibodies. **(B)** Immunoprecipitation (IP) of urinary EVs from a bladder cancer patient with a2M-specific antibody-conjugated beads (a2M). Rabbit IgG (IgG) was used as a negative control for precipitation. Input: urinary Evs from a bladder cancer patient before IP reaction. IP, protein lysate of immunoprecipitated urinary EVs with antibody-conjugated beads. Output: flow-through proteins from the IP reaction. The precipitated proteins were analyzed *via* immunoblotting with an anti-a2M or anti-CD63 antibody. The expected protein sizes are indicated by black arrows.

Several studies have reported the EV-mediated secretion of a2M (41–43). In western blot analysis, a2M was detected both in cell lysate and secreted EVs from BC cells (**Figure 6A**). Additionally, through immunohistochemistry of BC patient tissues, we detected a2M in the interstitial space as well as in tumor cells (**Figure 6B**), suggesting that a2M would originate from BC cells. To verify that a2M is an EV component, uEVs from BC patients were isolated *via* ExoDisc, followed by further separation through iodixanol gradients (**Figure 7A**). CD63, the two endosomal sorting complexes required for transport proteins (TSG101 and ALIX), and heat shock protein 70 (HSP70) were detected in the similar fractions as a2M. Immunoprecipitation of a2M with uEVs from BC patients demonstrated that CD63 was precipitated with a2M (**Figure 7B**).

## Discussion

To the best of our knowledge, this study is the first to demonstrate the diagnostic value of uEV-derived a2M for BC. Currently, no optimal workflow has been established for the analysis of uEVs (44). Thus, in order to identify clinically valuable uEV protein biomarkers, we first had to determine the optimal method for uEV isolation, while considering urine sample properties, such as inter-individual variation. Further, the validation of potential uEV markers in a clinical BC cohort is imperative. In the current work, we compared the yield of several existing EV isolation methods and analyzed the pre-and post-operative urinary EV protein levels in urine samples of actual BC patients in order to identify clinically relevant biomarkers. We then validated a2M as a non-invasive diagnostic biomarker in urine samples by analyzing urine from controls and BC patients. Through this approach, we determined that a2M in uEVs, but not in whole urine, was specific to BC patients and thus could indicate the presence of disease.

We first sought to identify an ideal urinary EV isolation method that can rapidly provide sufficient EV protein from a limited volume of urine samples for further experiments. While ultracentrifugation is considered the gold standard for EV isolation, it is not suitable for clinical use because it is time consuming, labor intensive, and requires specialized equipment (45, 46). Since there is no established standard technique for the isolation of uEVs from clinical samples of limited quantity, we compared the uEV yield between three different methods. ExoDisc provided a significantly greater yield of uEV protein than classical ultracentrifugation, consistent with a previous report (23). uEV protein quantification indicated that ultracentrifugation and ExoLutE were associated with a low yield of EV protein. In contrast, ExoDisc yielded sufficient uEV protein from as low as 4 ml of urine, which was then subjected to antibody array-based proteomics analysis, which enabled us to analyze uEV protein expression. Thus, uEV isolation *via* ExoDisc followed by antibody array-based proteomic analysis were adopted for further experiments.

uEV normalization is a topic of ongoing discussion (44). The most crucial point we considered was whether it was a normalization index that could be easily used in clinical practice. Urine creatinine may be a good one. However, since urine creatinine is an index before EVs isolation, it may not be reliable for the isolated EVs. We also considered the particle number of EVs. However, it would be difficult for the clinical lab to analyze it. In the current study, we used the quantity of protein as a normalization index, but this may not be the best. Therefore, we are also considering other normalization methods for better results, which is an area that needs more research.

We screened potential uEV protein biomarkers in patient urine, comparing their levels between pre- and post-operative uEVs from the same individual patient. Of note, this strategy has not been previously employed for the identification of uEV-based diagnostic biomarkers for BC. Several studies identified candidate uEV protein markers through proteomics analysis of the supernatant from BC cell lines and primary urothelial cells (19, 20). However, we believe that the cell culture supernatant does not recapitulate the uEV secretome observed in actual BC patients, as also reported by others (47). In addition, the heterogeneity between urine samples from BC patients should be considered, as established in previous studies (25–27). Taken together, our approach, namely the analysis of the uEV proteome between paired pre-and post-operative samples from the same patients, would be conducive for the identification of clinically relevant uEV markers in BC.

We determined that the expression of several proteins was significantly different between pre- and post-operative uEV samples. Notably, significant differences in cadherin-13, clusterin, and a2M levels were mainly observed in high-grade BC patients, while no significant difference was noted between the paired urine samples from low-grade BC patients. Although the exact reason for this finding remains unclear, high-grade BC patients may secrete more diverse proteins through uEVs when compared low-grade BC patients due to increased genetic instability (48, 49).

Among the three potential uEV proteins identified, we observed a pronounced difference in a2M levels, which we validated in a greater number of patients through ELISA (83 subjects in total). Furthermore, we demonstrated that uEV a2M expression in high-grade BC patients was significantly higher than that in low-grade BC patients. We also detected a2M in the whole urine samples of non-cancer controls, which decreased after extracting the uEVs from urine. This finding indicated that total urine a2M is not a robust biomarker for BC, in contrast to the uEV-specific a2M level. As over half of the subjects (52.2%, 12 out of 23) in the control group had hematuria, we concluded that the high expression of a2M in uEVs from BC patients was not due to hematuria. Furthermore, we determined that the diagnostic performance of a2M was superior to that of urine cytology, a well-established BC screening method (Youden index: 28.1% for a2M with a cut-off of 0.035 vs. 15.0% for urine cytology). Taken together, BC patients had significantly higher levels of uEV-associated a2M, which was rarely detected in non-cancer controls.

a2M, one of the large glycoproteins (720 kDa) present in bodily fluids, primarily functions as a protease inhibitor (28). a2M plays diverse and complex roles by binding to different hormones and regulating their activity; several studies on the association between a2M and cancer have indicated that the former might play an important anti-tumor role (50–52). A previous study suggested that a2M exerts anti-tumor effects by modulating tumor cell adhesion, migration, and growth (50). Other studies have shown that a2M bound to low-density lipoprotein receptor-related protein-1 inhibits the proliferation, migration, invasion, and growth of tumor cells (51, 52). To date, only one study has reported that a2M level is elevated in the serum of BC patients (53). In contrast, the expression of a2M in uEVs has not been assessed. Our current results highlighted the potential of uEV-derived a2M as a promising diagnostic marker for BC.

The limitation of this study is that we could not determine why the cells in patients with BC prefer to sort a2M into EVs. Presumably, as there is no significant difference in a2M levels in whole urine samples, the sorting of a2M into uEVs does not occur in non-cancer controls but selectively occurs in BC patients. Moreover, it is unidentified whether this phenomenon occurs in BC cells or other cells due to changes in the BC microenvironment. Further research is required to reveal the mechanism that causes selective a2M sorting into uEVs.

In conclusion, we demonstrated the potential of a2M as a novel uEV biomarker for BC diagnosis through an optimized EV isolation and analysis workflow. Our results highlight the diagnostic potential of uEV markers in BC. Further functional studies are needed to uncover the exact mechanisms of action of EV-associated a2M in BC pathophysiology; large-scale validation studies should be conducted in independent BC cohorts in order to confirm the diagnostic value of a2M in uEVs. We expect our approach for uEV isolation and analysis to serve as a practical basis for the identification of clinically useful uEV biomarkers for cancer diagnosis.

## Data availability statement

The original contributions presented in the study are included in the article/**Supplementary Material**. Further inquiries can be directed to the corresponding authors.

## Ethics statement

The studies involving human participants were reviewed and approved by The Institutional Review Board of Eulji University Hospital (No. 2018-07-010 and 2019-05-027-002). The patients/participants provided their written informed consent to participate in this study.

## Author contributions

M-SL and JP conceived the research design. JP and HP collected human urine samples. JL, SH, YK, JM, and H-WO conducted experiments and acquired data. DS performed statistical analyses. Y-KC advised on the study design and in preparing the manuscript. JL, JP, and M-SL wrote the main manuscript. All authors contributed to the article and approved the submitted version.

## Funding

This work was supported by the Basic Science Research Program through the National Research Foundation of Korea (NRF) funded by the Ministry of Education (NRF-2019R1I1A3A01060913 and NRF-2022R1F1A1065578 to JP), the Mid-career Research Program through the National Research Foundation of Korea (NRF-2019R1A2C2083947 to M-SL), and the KBRI basic research program through the Korea Brain Research Institute funded by the Ministry of Science and ICT (22-BR-01-03 to JM).

## Acknowledgments

We thank members of Lee’s laboratory for technical assistance and helpful discussions.

## Conflict of interest

Y-KC holds ExoDisc patents, which are licensed to LabSpinner (Ulsan, Korea).

The remaining authors declare that the research was conducted in the absence of any commercial or financial relationships that could be construed as a potential conflict of interest.

## Publisher’s note

All claims expressed in this article are solely those of the authors and do not necessarily represent those of their affiliated organizations, or those of the publisher, the editors and the reviewers. Any product that may be evaluated in this article, or claim that may be made by its manufacturer, is not guaranteed or endorsed by the publisher.
