# Supplementary material for: Alpha-2-macroglobulin as a novel diagnostic biomarker for human bladder cancer in urinary extracellular vesicles
Source: Front Oncol. 2022 Sep 13;12:976407. doi: 10.3389/fonc.2022.976407 (PMC9513419; doi:10.3389/fonc.2022.976407)
Supplement: Supplementary file 1 [file DataSheet_1.docx]

**Supplementary Table 1. Proteome profiler protein ID for human non-hematopoietic array**

| Coordinate | Analyte/Control | Alternate Nomenclature | Entrez Gene ID |
| --- | --- | --- | --- |
| A1,A2,A3,A4 | Reference Spots | N/A | - |
| A5, A6 | ADAM15 | N/A | 8751 |
| A7, A8 | βIG-H3 | N/A | 7045 |
| A9, A10 | BMPR-IB/ALK-6 | CDw293 | 658 |
| A11, A12 | Cadherin-4/R-Cadherin | CAD4 | 1002 |
| A13, A14 | Cadherin-11 | CAD11 | 1009 |
| A15, A16 | Cadherin-13 | CDHH | 1012 |
| A17, A18 | E-Cadherin | CD324, ECAD | 999 |
| A19, A20 | N-Cadherin | CD325, NCAD | 1000 |
| A23, A24 | Positive Control | N/A | - |
| B1, B2 | Positive Control | N/A | - |
| B5, B6 | P-Cadherin | PCAD | 1001 |
| B7, B8 | VE-Cadherin | CD144, CDH5 | 1003 |
| B9, B10 | Cathepsin D | N/A | 1509 |
| B11, B12 | CD40/TNFRSF5 | N/A | 958 |
| B13, B14 | CEACAM-5/CD66e | CEA | 1048 |
| B15, B16 | CHL-1/L1CAM-2 | CALL | 10752 |
| B17, B18 | Clusterin | N/A | 1191 |
| B19, B20 | Coagulation Factor II/Thrombin | N/A | 2147 |
| C1, C2 | COMP/Thrombospondin-5 | N/A | 1311 |
| C3, C4 | CRELD2 | N/A | 79174 |
| C5, C6 | Desmoglein 2 | N/A | 1829 |
| C7, C8 | ECM-1 | N/A | 1893 |
| C9, C10 | EGF R/ErbB1 | HER1 | 1956 |
| C11, C12 | Endoglycan | PODXL2 | 50512 |
| C13, C14 | EpCAM/TROP-1 | N/A | 4072 |
| C15, C16 | ErbB2/HER2 | N/A | 2064 |
| C17, C18 | ErbB3/HER3 | N/A | 2065 |
| C19, C20 | ErbB4/HER4 | N/A | 2066 |
| C21, C22 | ESAM | N/A | 90952 |
| C23, C24 | Galectin-2 | N/A | 3957 |
| D1, D2 | HPRG | N/A | 3273 |
| D3, D4 | Integrin α3/CD49c | ITGA3 | 3675 |
| D5, D6 | Integrin α5/CD49e | ITGA5 | 3678 |

| Coordinate | Analyte/Control | Alternate Nomenclature | Entrez Gene ID |
| --- | --- | --- | --- |
| D13, D14 | Jagged 1 | JAG1, CD339 | 182 |
| D15, D16 | JAM-B/VE-JAM | CD322, JAM2 | 58494 |
| D17, D18 | JAM-C | JAM3 | 83700 |
| D19, D20 | LRP-6 | N/A | 4040 |
| D21, D22 | MCAM/CD146 | MUC18 | 4162 |
| D23, D24 | MEPE | N/A | 56955 |
| E1, E2 | MUCDHL | N/A | 53841 |
| E3, E4 | Nectin-2/CD112 | PVRR2, PVRL2 | 5819 |
| E5, E6 | Nectin-4 | PVRL4 | 81607 |
| E7, E8 | Neurotrimin | IGLON2 | 50863 |
| E9, E10 | Notch-1 | N/A | 4851 |
| E11, E12 | NrCAM | N/A | 4897 |
| E13, E14 | Periostin/OSF-2 | N/A | 10631 |
| E15, E16 | Podocalyxin | PODXL | 5420 |
| E17, E18 | E-Selectin/CD62e | CD62E, ELAM | 6401 |
| E19, E20 | Semaphorin 3A | SEMA3A | 10371 |
| E21, E22 | SREC-I/SR-F1 | SCARF1 | 8578 |
| E23, E24 | SREC-II | SCARF2 | 91179 |
| F1, F2 | Stanniocalcin 1 | STC1 | 6781 |
| F3, F4 | Syndecan-1/CD138 | SDC1 | 6382 |
| F5, F6 | Syndecan-4 | SDC4 | 6385 |
| F7, F8 | Thrombospondin-2 | TSP2, THBS2 | 7058 |
| F9, F10 | TIMP-4 | N/A | 7079 |
| F11, F12 | TROP-2 | N/A | 4070 |
| F13, F14 | VAP-1/AOC3 | SSAO | 8639 |
| F15, F16 | VCAM-1 | CD106 | 7412 |
| F17, F18 | VEGF R1/Flt-1 | N/A | 2321 |
| F19, F20 | VEGF R2/KDR/Flk-1 | CD309 | 3791 |
| G1, G2 | Reference Spots | N/A | - |
| G5, G6 | IgM | N/A | 3507 |
| G7, G8 | α2-Macroglobulin | A2M | 2 |
| G9, G10 | Transferrin R | CD71 | 7037 |
| G11, G12 | Vimentin | VIM | 7431 |
| G13, G14 | PBS (Negative Control) | N/A | - |
| G23, G24 | Reference Spots | N/A | - |

**Supplementary Table 2. Proteome profiler protein ID for human common analytes array**

| Coordinate | Analyte/Control | Alternate Nomenclature | Entrez Gene ID |
| --- | --- | --- | --- |
| A1,A2,A3,A4 | Reference Spots | N/A _ | - |
| A5, A6 | ACE | CD143 | 1636 |
| A7, A8 | ADAM8 | CD156 | 101 |
| A9, A10 | ADAM9 | N/A | 8754 |
| A11, A12 | ADAM10 | CD156c | 102 |
| A13, A14 | ALCAM/CD166 | N/A | 214 |
| A15, A16 | Amphiregulin | AR | 374 |
| A17, A18 | APP (pan) | N/A | 351 |
| A19, A20 | BACE-1 | N/A | 23621 |
| A23, A24 | Reference Spots | N/A | - |
| B1, B2, B3, B4 | Positive Control | N/A | - |
| B5, B6 | BCAM | CD239 | 4059 |
| B7, B8 | C1q R1/CD93 | N/A | 22918 |
| B9, B10 | CD9 | N/A | 928 |
| B11, B12 | CD23/Fc ε RII | N/A | 2208 |
| B13, B14 | CD31/PECAM-1 | N/A | 5175 |
| B15, B16 | CD36/SR-B3 | FAT | 948 |
| B17, B18 | CD40 Ligand/TNFSF5 | CD154 | 959 |
| B19, B20 | CD44H | N/A | 960 |
| C1, C2 | CD58/LFA-3 | N/A | 965 |
| C3, C4 | CD90/Thy1 | N/A | 7070 |
| C5, C6 | CD99 | N/A | 4267 |
| C7, C8 | CD155/PVR | N/A | 5817 |
| C9, C10 | CEACAM-1/CD66a | N/A | 634 |
| C11, C12 | CX3CL1/Fractalkine | Neurotactin | 6376 |
| C13, C14 | CXCL8/IL-8 | NAP-1 | 3576 |
| C15, C16 | EMMPRIN/CD147 | BSG | 682 |
| C17, C18 | Endoglin/CD105 | N/A | 2022 |
| C19, C20 | Epiregulin | N/A | 2069 |
| C21, C22 | Galectin-1 | GAL1 | 3956 |
| C23, C24 | Galectin-3 | GAL3 | 3958 |
| D1, D2 | Galectin-3BP/MAC-2BP | N/A | 3959 |
| D3, D4 | HB-EGF | N/A | 1839 |
| D5, D6 | ICAM-2/CD102 | N/A | 3384 |

| Coordinate | Analyte/Control | Alternate Nomenclature | Entrez Gene ID |
| --- | --- | --- | --- |
| D7, D8 | IL-1 RII | CD121b | 7850 |
| D9, D10 | IL-15 Rα | N/A | 3601 |
| D11, D12 | Integrin β1/CD29 | ITGB1 | 3688 |
| D13, D14 | Integrin β2/CD18 | ITGB2 | 3689 |
| D15, D16 | Integrin β3/CD61 | ITGB3 | 3690 |
| D17, D18 | Integrin β4/CD104 | ITGB4 | 3691 |
| D19, D20 | Integrin β5 | ITGB5 | 3693 |
| D21, D22 | Integrin β6 | ITGB6 | 3694 |
| D23, D24 | JAM-A | CD321 | 50848 |
| E1, E2 | Lipocalin-2/NGAL | N/A | 3934 |
| E3, E4 | LOX-1/SR-E1 | CLEC8A | 4973 |
| E5, E6 | MD-1 | LY86 | 9450 |
| E7, E8 | MMP-2 (total) | N/A | 4313 |
| E9, E10 | NCAM-1/CD56 | N/A | 4684 |
| E11, E12 | NCAM-L1 | L1CAM, CD171 | 3897 |
| E13, E14 | Osteopontin | OPN | 6696 |
| E15, E16 | PAR1 | N/A | 2149 |
| E17, E18 | Pref-1/DLK-1/FA1 | N/A | 8878 |
| E19, E20 | RECK | N/A | 8434 |
| E21, E22 | Stabilin-1 | CLEVER-1, FEEL-1 | 23166 |
| E23, E24 | TACE/ADAM17 | CD156b | 6868 |
| F1, F2 | Thrombospondin | THBS, TSP | 7057 |
| F3, F4 | TIMP-1 | N/A | 7076 |
| F5, F6 | TIMP-2 | N/A | 7077 |
| F7, F8 | TIMP-3 | N/A | 7078 |
| F9, F10 | TNF RII/TNFRSF1B | CD120b | 7133 |
| G1, G2 | Reference Spots | N/A | - |
| G5, G6 | IgM | N/A | 3507 |
| G7, G8 | α2-Macroglobulin | A2M | 2 |
| G9, G10 | Transferrin R | CD71 | 7037 |
| G11, G12 | Vimentin | VIM | 7431 |
| G13, G14 | PBS (Negative Control) | N/A | - |
| G23, G24 | Reference Spots | N/A | - |
